# Energy Management Strategy for an Autonomous Hybrid Power Plant Destined to Supply Controllable Loads

**DOI:** 10.3390/s22010357

**Published:** 2022-01-04

**Authors:** Imene Yahyaoui, Natalia Vidal de la Peña

**Affiliations:** 1 Department of Applied Mathematics, Materials Science and Engineering and Electronic Technology, University Rey Juan Carlos, 28933 Madrid, Spain; 2 Chemical Engineering Department, Faculty of Applied Sciences, University of Liège, 4000 Liège, Belgium; nvidal@uliege.be

**Keywords:** photovoltaic energy, wind energy, batteries state of charge, diesel generator, fuzzy logic, energy management

## Abstract

This paper proposes an energy management strategy (EMS) for a hybrid stand-alone plant destined to supply controllable loads. The plant is composed of photovoltaic panels (PV), a wind turbine, a diesel generator, and a battery bank. The set of the power sources supplies controllable electrical loads. The proposed EMS aims to ensure the power supply of the loads by providing the required electrical power. Moreover, the EMS ensures the maximum use of the power generated by the renewable sources and therefore minimizes the use of the genset, and it ensures that the batteries bank operates into the prefixed values of state of charge to ensure their safe operation. The EMS provides the switching control of the switches that link the plant components and decides on the loads’ operation. The simulation of the system using measured climatic data of Mostoles (Madrid, Spain) shows that the proposed EMS fulfills the designed objectives.

## 1. Introduction

Microgrids can be classified as grid-connected or stand-alone. It can also be classified following the use that can be for military, industrial, residential, or agricultural applications, etc. In addition, these microgrids can be based on renewable sources, namely, photovoltaic panels, wind turbines, fuel cells, waves energy, etc., or nonrenewable energy sources, namely, gensets. Therefore, the diversity of microgrids results in different architectures that are managed following a specific management strategy that fulfills the goals, the types of the power sources, and the loads (controllable/noncontrollable).

For the case of renewable stand-alone plants, many studies have been developed and focused on the energy management. A literature review shows that the energy management problems related to different fields of microgrids have been solved by various algorithms. For instance, in [1], the authors developed an intelligent control strategy based on the ANN and applied on a grid-connected plant composed of Photovoltaic (PV) panels and a Li-ion Battery Energy Storage System. The ANN is used to estimate the battery bank state of charge (SOC), which is used to decide on the battery bank charging and discharging using a smart controller for a DC/DC bidirectional converter.

The same technique (ANN) is applied in [2]. In fact, the authors developed a Home Energy Management System (HEMS) based on the ANN for the home appliances scheduling and efficient use of the storage elements.

In [3], the ANN is mixed with the Particle Swarm Optimization (PSO) technique and used for the microgrid optimal energy scheduling. In fact, in this paper, the authors proposed an enhancement for the (ANN) using the PSO to manage renewable energy resources (RESs) in a virtual power plant (VPP) system. The authors reached the optimum number of neurons in the hidden layers of the ANN and then obtained a more accurate EMS.

Multi objective algorithms have also been used for the energy management of microgrids. For instance, in [4], the authors used a tri-objective optimization framework for the energy management of smart homes based on the demand response. The EMS includes the operating cost, emissions, and peak-to-average ratio.

The research paper [5] focused on the use of the stochastic multi-objective for the optimal energy management of grid-connected unbalanced microgrids with renewable energy generation and plug-in electric vehicles. In this paper, a stochastic multi-objective optimization model for grid-connected unbalanced MGs is applied to minimize the total operational cost, energy management, and control the voltage. In [6], the authors investigated the problem of the day-ahead operation of a grid-connected MG, integrated with distributed generation units and storage systems. For this, a θ-modified krill herd approach is employed to solve the problem of energy management and cost minimization.

In addition to the previous method used in the literature for the energy management for renewable plants, fuzzy logic is also used for energy management in renewable autonomous plants. The good efficiency of this tool is given by its ease of use and the correct results that it gives [7]. In fact, fuzzy logic is a mathematical theory of fuzzy set [8,9,10]. Suganthi et al. define fuzzy logic as follows: “Fuzzy logic deals with reality, and it is a form of much valued logic” [11]. Michael and Warne considered the fuzzy logic as follows: “a method of readily representing human expert knowledge on a digital processor in particular where mathematical or rule-based expert systems experience difficulty” [12].

Thus, fuzzy logic is used in energy management research works since it allows for the possibility to describe systems behaviors and to make control decisions using linguistic rules, based on the expert knowledge. Moreover, the fuzzy rules are written using a simple linguistic manner, which describes the adopted approach in taking control decision. This makes it an easy tool for making decisions. For example, in [13], the authors used the fuzzy logic to control the charge and discharge of multiple types of storage systems in a hybrid plant. Indeed, measured climatic data collected from the University of Queensland (Australia) are used to test the fuzzy logic efficiency. Therefore, the power flow is controlled, and the excess power generated is used to charge the lithium batteries. The energy management strategy is tested using multiple operating scenarios, and the obtained results confirmed its capability to control the hybrid renewable energy system.

Moreover, in [14], the authors applied the fuzzy logic to control the power flow in a hybrid plant composed of wind generators, decentralized generators, and storage systems for the production of electrical power. The controller is tested using a benchmark plant installed in the laboratory. The fuzzy logic is used to decide on the charging of the storage system elements and the frequency control.

Derrouazin et al. used fuzzy logic multi-input-output to manage the energy flux of a hybrid system with a solar photovoltaic, wind turbine, and battery. The obtained results successfully show the signals of the electronic switches based on the input power states of the hybrid power system [15].

Moreover, some software linked to the renewable energies are also developed. For example, PVSyst [16] is used to size the PV plants components (autonomous, connected to the grid, or destined to PV water pumping). The General Algebraic Modelling System (GAMS) is a modelling system that uses optimization techniques to solve mathematical and optimization problems [17]. HOMER [18] is a software that is widely used to optimize the hybrid renewable system based on economic criteria. This software allows one to deduce the operating of the power sources that generate the electrical power required by the load. Hence, it solves a cost function based first on economic criteria.

**Figure 1:**
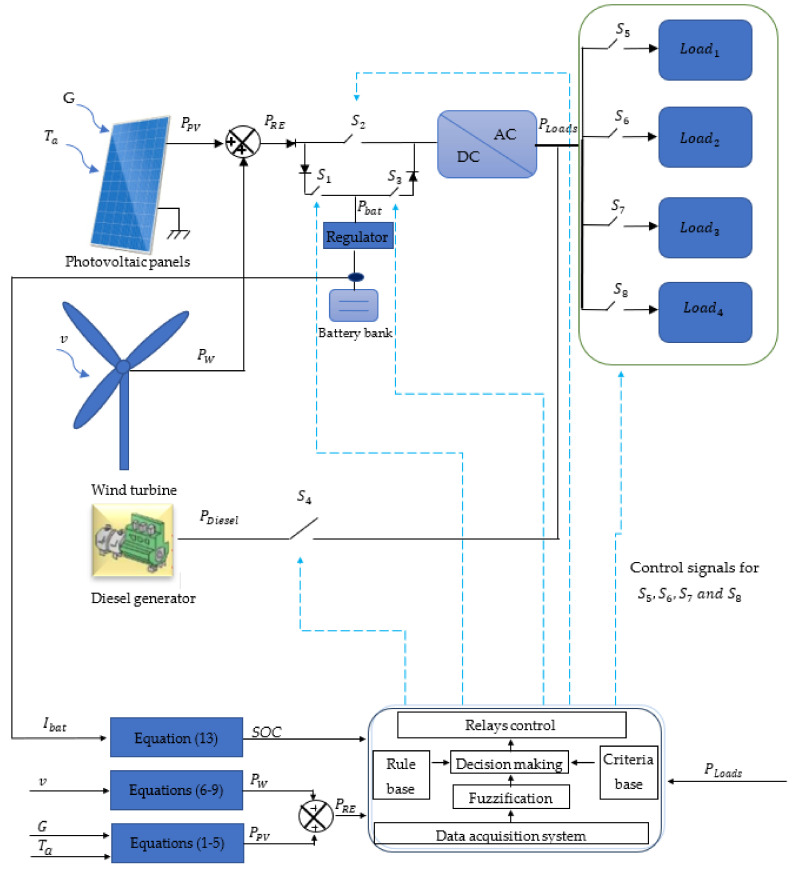
The bloc scheme of the studied hybrid RE plant.

This paper is a continuation of previous published papers [19,20]. In fact, it focuses on the energy management strategy (EMS) of an autonomous hybrid plant destined to supply controllable loads with the required electrical power. The studied plant is composed of PV panels, a wind turbine, a genset, and a battery bank, which are connected via controllable switches (Figure 1). More precisely, the EMS aims to decide on the switching of the switches following an EMS, which also decides the load(s) operation.

Therefore, in this paper, the authors focus on the EMS using the fuzzy logic, since the expert (the user) can decide the management strategy in a linguistic way, which is very easy to program and implement. In previous papers [19], some of the authors of this paper have studied the energy and water management of a renewable based plant. Moreover, in [20], the authors studied the optimization of a hybrid plant using the genetic algorithm. The contributions of this paper are the following:

➢ The authors apply the fuzzy logic to decide on the switching time of the switches that links the hybrid plant’ components (Figure 1). For this, an EMS is designed and tested using the measured climatic data of Mostoles, (Madrid, Spain), during three typical days of July, March, and December.
➢ The load operation is also deduced using the EMS.
➢ The EMS is applied in a hybrid plant with the objective of maximizing the use of the power generated from the renewable energy components, on the one hand, and minimizing the use of the battery bank and especially the diesel generator, on the other one.

The paper is organized as follows: Section 2 describes the materials and the methods, where the management strategy and the algorithm are explained in depth. In Section 3, the results are represented. Then, discussions are detailed in Section 4. Finally, Section 5 provides the conclusions.

## 2. Materials and Methods

### 2.1. System Components Modeling

As has previously been described, the hybrid plant is composed of a set of photovoltaic panels (equipped with MPPT controllers), a wind turbine, and a genset, which are connected through switches to a Lead-acid battery bank (that includes the batteries and their regulators) and set of electrical loads, as presented in Figure 1. In the following Section 2.1.1, the components models used in this research paper are detailed:

#### 2.1.1. Photovoltaic Power Generation Model

A nonlinear model is used to describe the power generated by the PV panels $P_{PV}$, which depends basically on the measured radiation *G* and the ambient temperature $T_{a}$. The nonlinear model allows the generated current $I_{pv}$ to be obtained (Equations (1)–(5)). Then, the power $P_{PV}$ is obtained by multiplying the current by the voltage [21,22,23]:

(1) $$I_{pv}=n_{p}\left(I_{ph}-I_{r}\left(\exp\;\left(\frac{V_{c}+I_{PV}R_{s}}{V_{t\_T_{a}}}\right)-1\right)\right)$$

(2) $$I_{ph}=\frac{G}{G_{0}}I_{sc}$$

(3) $$I_{sc}=I_{sc\_T_{ref}}\left(1+\left(a\;\left(T_{a}-T_{ref}\right)\right)\right)$$

(4) $$I_{r}=I_{r\_T_{ref}}{\left(\frac{T_{a}}{T_{ref}}\right)}^{\frac{3}{n}}e^{\left(\frac{-qV_{g}}{nK}\left(\frac{1}{T_{a}}-\frac{1}{T_{ref}}\right)\right)}$$

(5) $$I_{r\_T_{ref}}=\frac{I_{sc\_T_{ref}}}{e^{\frac{qV_{c}}{nKT_{ref}}}-1}$$

where:

$I_{pv}$: the estimated photovoltaic current (A),
$I_{ph}$: the generated photo-current at a given irradiance *G* (A),
$I_{sc}$: the short circuit current for a given temperature $T_{a}$ (A),
$I_{r}$: the reverse saturation current for a given temperature $T_{a}$ (A),
$I_{r\_T_{ref}}$: the reverse saturation current for the reference temperature $T_{ref}$ (A),

Then, a P&O MPPT bloc is used to track the maximum power point (MPP).

#### 2.1.2. Wind Turbine Generation Model

Wind turbines uses part of the kinetic energy of the wind to produce electrical power [24,25]. The wind turbine power $P_{W}$ is obtained using a non- linear equation, which depends on the wind speed *v*, the design of the wind turbine (namely, the radius of the wind turbine), and the pitch angle β.

(6) $$P_{W}=\frac{1}{2}\rho \;A\;C_{p}\;\left(\lambda_{1}\right)\;\;v^{3}$$

where ρ is the air density (kg·m^−3^), A is the surface of the turbine blades (m^2^), and $C_{p}$ is the power coefficient [24,25]:

(7) $$C_{p}\left(\lambda_{1}\right)=0.5176\;\;\left(\frac{116}{\lambda_{i}}-0.4\beta -5\right)\;e^{\frac{-21}{\lambda_{i}}}+0.0068\;\;\lambda_{1}$$

(8) $$\frac{1}{\lambda_{i}}=\frac{1}{\lambda_{1}+0.08\beta }-\frac{0.035}{\beta^{3}+1}$$

and:

(9) $$\lambda_{1}=\frac{\Omega \;R}{v}$$

where: R is the wind turbine radius (m) and Ω is the angular mechanic speed (rad·s^−1^).

#### 2.1.3. Diesel Generator Model

**Figure 2:**
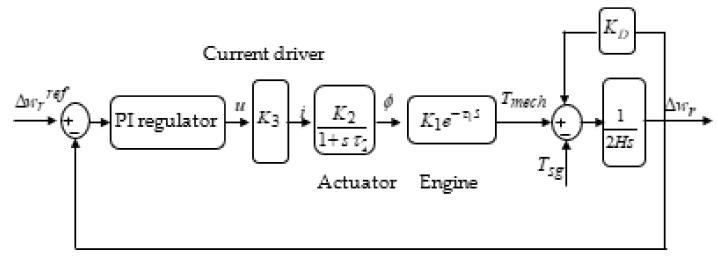
Diagram of the diesel engine components.

The diesel engine system is composed of the current driver, the actuator, the engine, and the flywheel (Figure 2) [26,27].

In fact, the current driver, which is presented by a constant $K_{3}$, is responsible for generating the mechanical torque by actuating on the fuel, which is injected into the combustion chamber and is therefore used to automatically control the engine speed. In the Engine block, the injected fuel is ignited by the compressed hot air in the combustion chamber, causing the movement of the piston during the power strokes, and the mechanical torque $T_{mech}$ is therefore produced, which is given by the following expression [26,27]:

(10) $$2H\frac{d\Delta w_{r}}{dt}=T_{mech}-T_{sg}-K_{D}\Delta w_{r}$$

(11) $$\frac{d\delta }{dt}=w_{0}\Delta w_{r}$$

(12) $$\Delta w_{r}=w_{r}-1$$

where the time *t* is the time (s), δ is the rotor angle (radian), $w_{0}$ is the rated generator speed (rad/s), $\Delta w_{r}$ is the speed deviation (pu), $w_{r}$ is the rotor angular velocity (pu), $T_{sg}$ is the generator torque (pu), and H is the inertia constant.

#### 2.1.4. Battery Bank Model

For the Lead-acid battery modelling, the nonlinear model studied by [28,29,30] is used. In fact, in this model, the battery is modeled by its State of Charge (SOC), which is given by the following equation:

(13) $$SOC=\frac{E_{bat\;charged}}{N_{bat}\times C_{bat}{}^{k_{p}}\times V_{bat}}$$

where:

$E_{bat\;charged}$: the available energy in the battery bank (Wh),
$N_{bat}$: the batteries number,
$C_{bat}$: battery’s nominal capacity (Ah),
$k_{p}$: Peukert constant,
$V_{bat}$: battery bank nominal voltage (V).

#### 2.1.5. Loads Model

In this paper, four loads are used. For this, their respective nominal powers are considered, which are: $P_{\mathrm{Load}\;1}=1000\;W$, $P_{\mathrm{Load}\;2}=2000\;W$, $P_{\mathrm{Load}\;3}=3000\;W$, and $P_{\mathrm{Load}\;4}=4000\;W$.

### 2.2. Fuzzy Management Algorithm (FMA)

Once the components’ models are explained, then it is possible to determine the fuzzy management algorithm. In fact, first the management strategy is detailed, which allows the switching modes to be obtained. Then, the fuzzy management strategy is explained in depth.

#### 2.2.1. Management Strategy

The management strategy follows these objectives:

1. The load must be supplied with stability. For this:
  - The renewable energy sources supply the loads (without the battery bank participation) only when the power demanded by the loads is lower than the power generated by the RE sources.
  - The time between switches of the switches is controlled. Consequently, the switching of the switches is minimized.
2. The battery bank is used to save the unused RE, which is later used by the loads during the night or when the renewable power generated is less than the power required by the controllable loads.
3. To protect the batteries against deep discharges and excessive charges. For this, during the charging or discharging process, the *SOC* should be maintained between the following limits ($SOC_{minimum}$ = 10% and $SOC_{maximum}$ = 90%).
4. The battery bank can only be charged by the power generated by the renewable sources $P_{RE}$.

In addition, as this paper manages the loads operation, an additional management strategy for the loads switching has also been designed. In fact, since the RE plant is equipped with four controllable loads connected to the power sources via four controllable switches $S_{5},\;S_{6},\;S_{7}$, and $S_{8}$, it is necessary to design a management strategy that decides which load(s) should be operating. In fact, the load management strategy is designed following the following objectives:

- Deducing the time and identifying which load(s) should be operating, depending on the power demanded.
- Minimizing the switching of the loads.

#### 2.2.2. Switching Modes

The management strategy of the generated power is based on connecting/disconnecting the three switches $S_{1}$, $S_{2}$, and $S_{3}$, depending on fuzzy rules that use the generated renewable power, the SOC, and $P_{Loads}$ as inputs and generates the signals control for the switches.

On the other hand, for the management strategy of the load’s operation, the switches $S_{5},\;\;S_{6},\;S_{7}$, and $S_{8}$ are used to connect and disconnect the loads (Figure 1).

Therefore, for the power provided for the loads, six switching modes can be identified, based on the logical combinations of switches.

- Mode 1: During this mode, only the switch $S_{1}$ is switched ON. The battery is moderately or almost discharged. Thus, all the renewable power $P_{RE}$ is used to feed the battery.

In this mode,

(14) $${\overset{⁻}{P}}_{Bat}=P_{RE}$$

- Mode 2: The switches $S_{1}$ and $S_{2}$ are switched ON. In this case, the RE sources provide the power required by the loads. If the batteries are not fully charged, the excessed RE power is used to charge the battery bank. Otherwise, this energy is not used.

In this mode,

(15) $$P_{pump}=P_{RE}$$

- Mode 3: In this mode, the switches $S_{2}$ and $S_{3}$ are switched ON, while $S_{1}$ is switched OFF. In this mode, the battery bank is moderately or fully charged. Thus, it supplies the loads together with the photovoltaic panels and the wind turbine with the required electrical power. Indeed, it is common to have this mode during the earliest hours of the morning or the latest hours of the afternoon during which the RE power is not sufficient for supply the loads with the required power. Hence, the battery bank provides the loads with the remaining power needed.
  In this mode,
  (16) $$P_{Loads}=P_{RE}+{\overset{⁻}{P}}_{Bat}$$
- Mode 4: This mode is possible during the night. In this mode, only $S_{3}$ is switched ON. In this case, the battery moderately or fully charged supplies the loads.

In this mode,

(17) $$P_{Loads}={\overset{⁻}{P}}_{Bat}$$

- Mode 5: This mode is obtained in the case that the power generated by the RE sources is not sufficient and the battery bank is totally discharged or in the case that there is no power required by the load. Hence, the renewable energy is used to charge the batteries and the loads are supplied by the diesel generator. Therefore, the switches $S_{1}$ and $S_{4}$ are switched ON, while the switches $S_{2}$ and $S_{3}$ are switched OFFOFF.
- Mode 6: This mode is activated when only the switch $S_{2}$ is ON, while the rest of the switches are OFF.

As it has already been explained before, the operation of the loads is also performed via the loads management strategy. For this, four loads have been used for this purpose. Indeed, Loads 1, 2, 3, and 4 have the following nominal powers, respectively: 1000 W, 2000 W, 3000 W, and 4000 W.

The switching of the switches that connect the loads is deduced following the following strategy:

If $P_{Loads}\in \left[10\;W\;1000\;W\right]$, then Load 1 is ON, Load 2 is OFF, Load 3 is OFF, and Load 4 is OFF.

If $P_{Loads}\in \left[1000\;W\;2000\;W\right]$, then Load 1 is OFF, Load 2 is ON, Load 3 is OFF, and Load 4 is OFF.

If $P_{Loads}\in \left[2000\;W\;3000\;W\right]$, then Load 1 is OFF, Load 2 is OFF, Load 3 is ON, and Load 4 is OFF.

If $P_{Loads}\in \left[3000\;W\;4000\;W\right]$, then Load 1 is OFF, Load 2 is OFF, Load 3 is OFF, and Load 4 is ON.

If $P_{Loads}\in \left[4000\;W\;5000\;W\right]$, then Load 1 is OFF, Load 2 is ON, Load 3 is ON, and Load 4 is OFF.

If $P_{Loads}\in \left[5000\;W\;6000\;W\right]$, then Load 1 is OFF, Load 2 is ON, Load 3 is OFF, and Load 4 is ON.

If $P_{Loads}\in \left[6000\;W\;7000\;W\right]$, then Load 1 is OFF, Load 2 is OFF, Load 3 is ON, and Load- 4 is ON.

### 2.3. Fuzzy Management Algorithm

Since the management strategy requires the previous knowledge of the system, the proposed management algorithm is based on fuzzy logic, as it requires previous knowledge of the process. Therefore, in this paper, the fuzzy management algorithm (FMA) is done in four steps [31,32,33,34]: the knowledge base, the fuzzification, the inference diagram, and the defuzzification, which are described now in detail.

#### 2.3.1. The Knowledge Base

**Figure 3:**
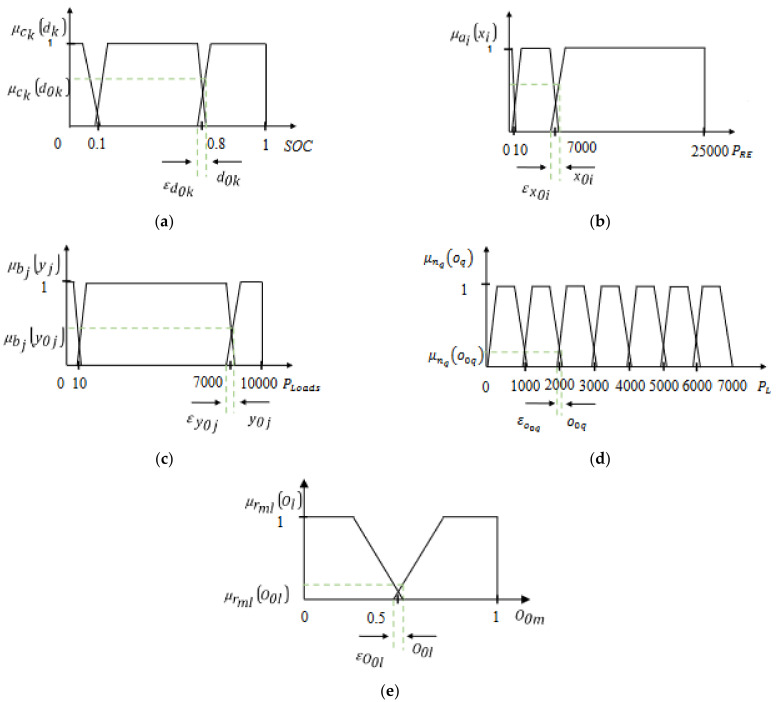
The membership functions corresponding to: (**a**) RE power $P_{RE}$, (**b**) Battery *SOC*, (**c**) Power demand $P_{Loads}$, (**d**) Power each load $P_{L}$, and (**e**) Switching orders of the relays.

The knowledge base is considered the first step of the FMA. In fact, five fuzzy trapezoidal partitions are used (Figure 3):

- First partition (Renewable power $P_{RE}$)

The first partition is defined by three fuzzy sets nominated $a_{i}$ = (A, B, C). These subsets cover the interval *x* = [0, 7000 W] and verify:

(18) $$\forall \;x_{i}\;\in \;x,\;\overset{3}{\underset{i=1}{\sum }}\;\mu_{a_{i}}\;\left(x_{i}\right)\;=1$$

where $\mu_{a_{i}}\left(x_{i}\right)$ is the membership function corresponding to $a_{i}$ evaluated at $x_{i}$.

- Second partition (State of Charge SOC)

The second partition is used to manage the SOC. Indeed, it is composed of three fuzzy sets, which are $c_{k}$ = (X, Y, Z). The interval d = [0, 1] is covered by these three sets and verify:

(19) $$\forall \;d_{k}\;\in \;d,\overset{3}{\underset{k=1}{\sum }}\;\mu_{c_{k}}\;\left(d_{k}\right)\;=1$$

where $\mu_{c_{k}}\left(d_{k}\right)$ is the membership function corresponding to $c_{k}$ evaluated at $d_{k}$.

- Third partition (total required loads’ power $P_{Loads}$)

The third partition is designed by three fuzzy sets $b_{j}=\left(D,E,F\right)$. The interval y = [0, 7000 W] must be covered by these three fuzzy sets and verify:

(20) $$\forall \;y_{j}\;\in \;y,\overset{3}{\underset{j=1}{\sum }}\;\mu_{b_{j}}\;\left(y_{j}\right)\;=1$$

where $\mu_{b_{j}}\left(y_{j}\right)$ is the membership function corresponding to $b_{j}$ evaluated at $y_{j}$.

- Fourth partition (Loads’ operation)

To decide on which load(s) should be operating, depending on the total power demanded by the loads, a fourth partition is designed using the following sets $n_{q}=\left(G,H,I,J,K,L,M\right)$. The interval o = [0, 4000 W] must be covered by these seven fuzzy sets and verify that:

(21) $$\forall \;o_{q}\;\in \;o,\overset{7}{\underset{q=1}{\sum }}\;\mu_{n_{q}}\;\left(o_{q}\right)\;=1$$

where $\mu_{n_{q}}\;\left(o_{q}\right)$ is the membership function corresponding to $n_{q}$ evaluated at $o_{q}$.

- Fifth partition (switches $R_{1},\;R_{2},\;R_{3},\;R_{4},\;R_{5},\;R_{6},\;R_{7}\;and\;R_{8}$

For controlling the switches, two fuzzy sets are used, which are the following: $r_{ml}$ = (V, W) cover the domain *O* = [0, 1] and verify:

(22) $$\forall \;O_{l}\;\in \;\;O,\overset{2}{\underset{l=1}{\sum }}\;\mu_{r_{ml}}\;\left(O_{l}\right)\;=1$$

where $O_{l}$ is the switching signal given to the switches to connect or disconnect the RE sources, the battery, and the diesel generator to the loads (Figure 1). $\mu_{r_{ml}}\left(O_{l}\right)$ is the membership function corresponding to $r_{ml}$ evaluated at $O_{l}$.

Therefore, to optimize the switching time of the switches, the fuzzy rules are classified according to three intervals of SOC:

❖ First case: SOC ∈  X = [0, 0.1]: in this interval, charging the battery is preferred to supplying the loads.
❖ Second case: SOC ∈  Y = [0.1, 0.8]: in this interval, supplying the loads is preferred to charging the battery bank.
❖ Third case: SOC ∈  Z = [0.8, 1]: in this case, the loads are supplied by the RE sources and/or the battery.

#### 2.3.2. Fuzzification

The fuzzification step consists in computing the membership functions $\mu_{a_{i}}\left(x_{0i}\right)$, $\mu_{c_{k}}\left(d_{0k}\right)$, $\mu_{b_{j}}\left(y_{0j}\right)$, $\mu_{n_{q}}\;\left(o_{q}\right)$, and $\mu_{r_{ml}}\left(O_{0l}\right)$ using symmetric trapezoidal shapes (Figure 3).

Mathematically, these membership functions are expressed by:

(23) $$\mu_{a_{i}}\left(x_{0i}\right)=\left\{\frac{x-x_{0i}}{\varepsilon_{x_{0i}}}if⥂\left|x-x_{0i}\right|\leq \varepsilon_{x_{0i}}else=0\right\}$$

which is used for the RE power $P_{RE}$,

(24) $$\mu_{c_{k}}\left(d_{0k}\right)=\left\{\frac{d-d_{0k}}{\varepsilon_{d_{0k}}}if⥂\left|d-d_{0k}\right|\leq \varepsilon_{d_{0k}}else=0\right\}$$

used for the battery State of Charge SOC,

(25) $$\mu_{b_{j}}\left(y_{0j}\right)=\left\{\frac{y-y_{j}}{\varepsilon_{y_{0j}}}if⥂\left|y-y_{0j}\right|\leq \varepsilon_{y_{0j}}else=0\right\}$$

which is considered for the required power by the load,

(26) $$\mu_{n_{q}}\;\left(o_{q}\right)=\left\{\frac{o-o_{q}}{\varepsilon_{o_{0q}}}if⥂\left|o-o_{0q}\right|\leq \varepsilon_{o_{0q}}else=0\right\}$$

which is considered for the operating load(s),

(27) $$\mu_{r_{ml}}\left(O_{0l}\right)=\left\{\frac{O-O_{0l}}{\varepsilon_{O_{0l}}}if⥂\left|O-O_{0l}\right|\leq \varepsilon_{O_{0l}}\;else=0\right\}$$

which is used for the switching orders of the switches,

$x_{0i}$, $d_{0k}$, $y_{0j}$, $o_{0q}$, and $O_{0l}$, which are, respectively, the values of the variables x, d, y, o, and O in the membership intervals; and $\varepsilon_{x_{0i}}$, $\varepsilon_{d_{0k}}$, $\varepsilon_{y_{0j}}$, $\varepsilon_{o_{0q}}$, and $\varepsilon_{O_{0l}}$ are the range values of $x_{0i}$, $d_{0k}$, $y_{0j}$, $o_{0q}$, and $O_{0l}$, respectively.

#### 2.3.3. Inference Diagram

The inference diagram is performed using following equation [35,36,37,38]:

(28) $$S_{ikjql}:if\;x_{i}\;is\;a_{i}\;and\;d_{k}\;is\;c_{k}\;and\;y_{j}\;is\;b_{j}\;and\;o_{q}\;is\;n_{q}\;then\;O\;is\;r_{l}$$

where *x_i_* corresponds to the RE power, *c_k_* is for SOC, *y_j_* is for the power by the loads P_Loads_, $o_{q}$ is for the load(s) operating, and ($a_{i}$, $c_{k}$, $b_{j}$, $o_{q}$) are, respectively, their linguistic values. *O* is the control signal for the switches, such that each switch receives a different control signal, knowing that $S_{l}$ is its linguistic value.

#### 2.3.4. Defuzzification

The output $O_{0l\;\left(l=1,\;2,\;3,\;..,8\right)}$ is calculated using the centroid method for defuzzification, which is performed using the following equation:

(29) $$O_{0l}=\frac{\int_{0}^{1}O_{l}\mu_{rl}d_{O_{l}}}{\int_{0}^{1}\mu_{rl}d_{O_{l}}}$$

Thus, the control of the seven switches is performed as follows:

(30) $$if\;O_{0l}≺\;0.5,\;\;then\;\;S_{l}=OFF\;,\;if\;O_{0l}\geq \;\;0.5,\;\;then\;\;S_{l}=ON$$

### 2.4. Algorithm Execution

The FMA is performed using the real-time execution of the algorithm following these steps:

1. Estimation of the RE power generation based on the measured solar radiation, the ambient temperature, and the wind speed.
2. Estimation of the demanded power $P_{Loads}$
3. Estimation of the *SOC* based on the measured battery current.
4. Fuzzification of the fuzzy inputs.
5. Inference diagram performance.
6. Defuzzification: Deduction of the fuzzy outputs $O_{l}$ using the trapezoidal method.

## 3. Results

The EMS allows the operating of the plant components to be deduced in such a way that the RE sources and/or the battery bank and the diesel generator supply the loads with AC power. Moreover, the operation of the loads is deduced since, depending on the power required by the four loads, the EMS decides also on which load(s) should be operating.

**Figure 4:**
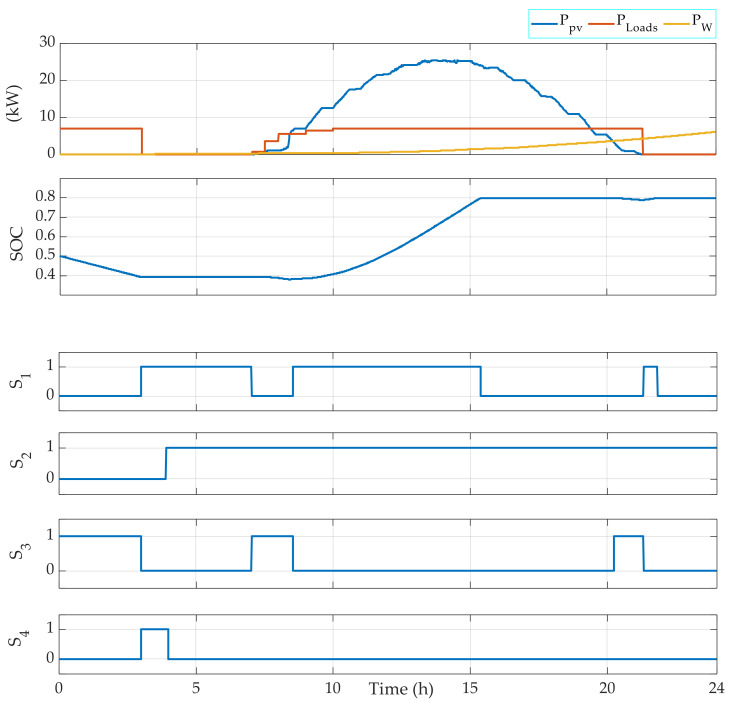
EMS results obtained for a typical day in July with an initial SOC = 50%.

**Figure 5:**
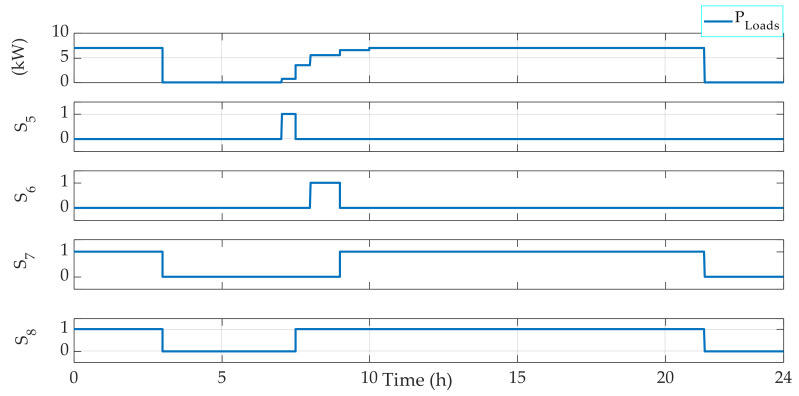
Loads energy management results.

**Figure 6:**
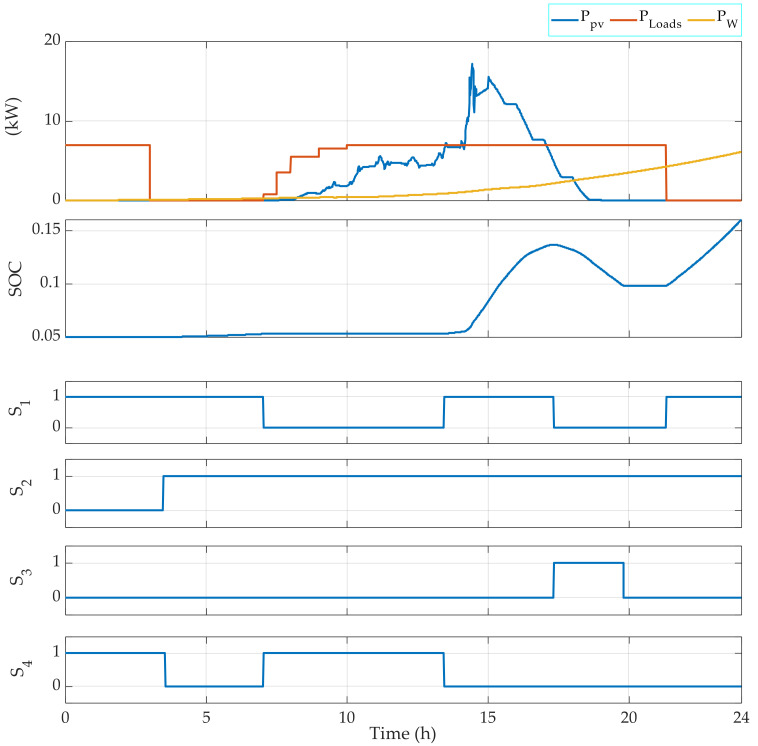
EMS results for the photovoltaic system in March using a variable loads power using an initially fully discharged battery bank.

**Figure 7:**
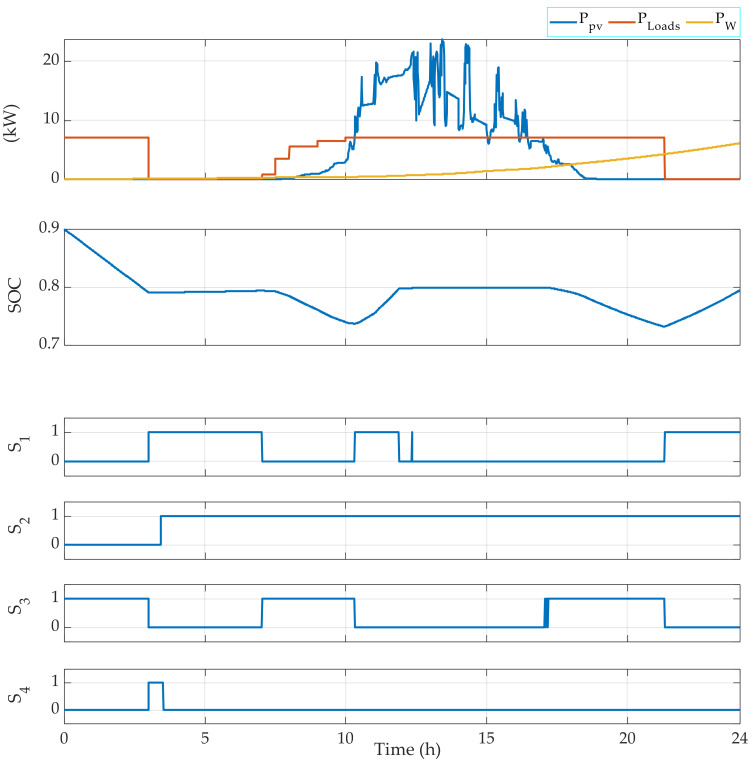
EMS results for the photovoltaic system in March using a variable loads power using an initially fully charged battery bank.

**Figure 8:**
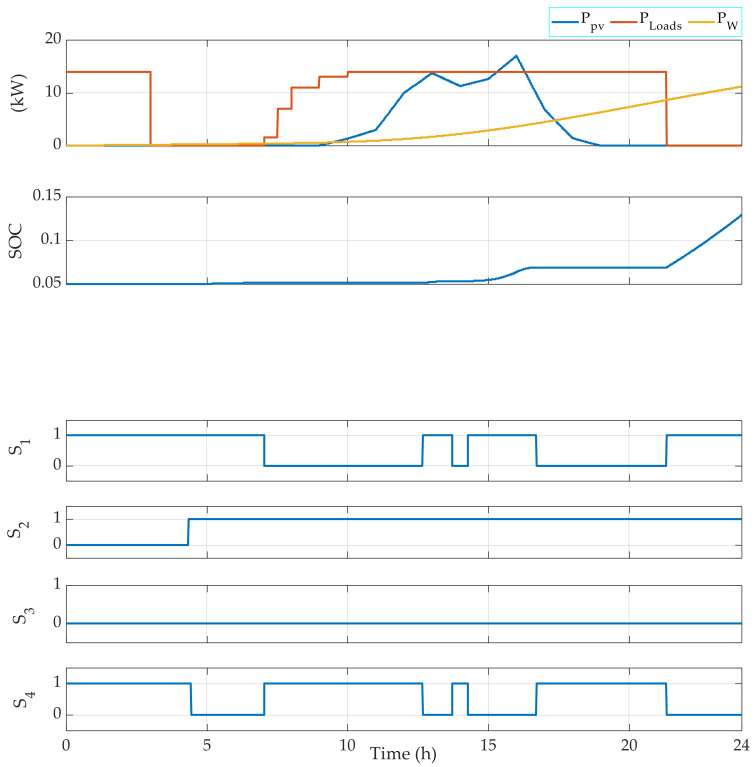
EMS results for the photovoltaic system in December using a variable loads power using an initially fully discharged battery bank.

**Figure 9:**
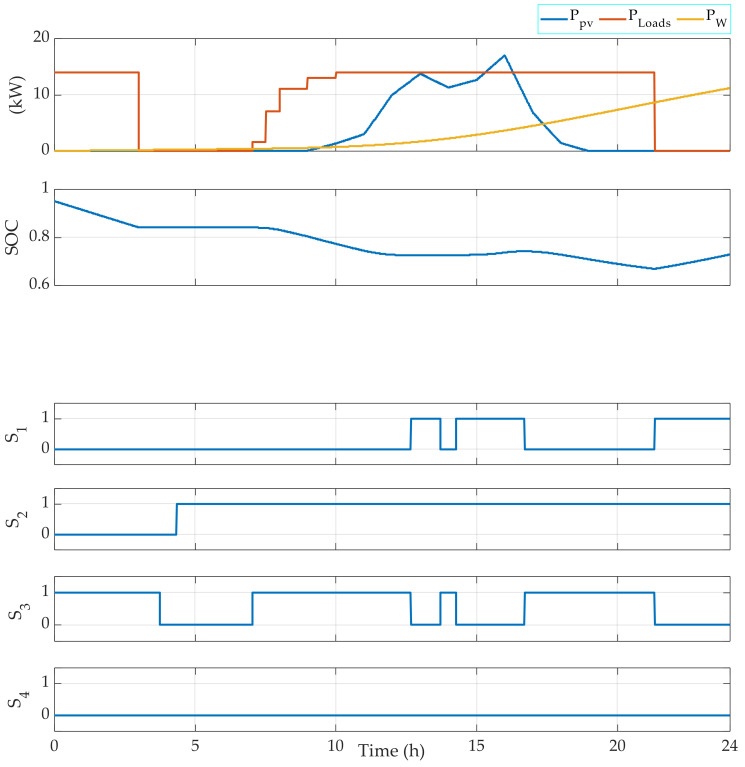
EMS results for the photovoltaic system in December using a variable loads power using an initially fully charged battery bank.

For this, measured climatic data of the solar radiation, the ambient temperature, and the wind speed that correspond to a typical day in July, March, and December have been used to test the FMA efficiency. The results are given in Figure 4, Figure 5, Figure 6, Figure 7, Figure 8 and Figure 9.

Figure 4 represents the results obtained by applying the EMS using climatic data that correspond to a typical day in July. The figure shows a correct operating of the switches and a safe operating of the battery banks, since the SOC is maintained between 0.1 and 0.8. Moreover, the diesel is operating only when the renewable power and the battery bank are not able to generate the power required by the loads. The renewable power is used to charge the battery bank in the case that the battery bank is not fully charged.

Secondly, the loads energy management is also tested. In fact, a variable profile of the power required by the loads is used. The profile is designed using four loads of 1000 W, 2000 W, 3000 W, and 4000 W. Hence, the FMA also allows one to decide which load(s) should be operating based on the total power required by the loads (Figure 5).

Figure 6 shows that the FMA gives good results during a typical day of March, during which there are fluctuations in the power generated by the renewable sources. In fact, in this simulation and to perform all the possible operating modes, the battery banks are considered discharged. Therefore, the switch $S_{3}$ is switched ON only when the SOC reaches the minimum allowed (10%). Otherwise, the diesel generator and the renewable sources are used to generate the required power.

The FMA has also been tested considering a charged battery bank during a day of March. Therefore, the initial SOC considered is 90%. The results are presented in Figure 7.

Figure 8 summarizes the FMA results for a typical day in December with the specific case of a battery bank initially discharged. In fact, at midnight, since the battery bank is initially discharged, the switches $S_{1}$ and $S_{4}$ are switched ON. Therefore, the renewable energy is used to charge the battery bank, whereas the genset is used to generate the power required by the loads. This corresponds to Mode 5. Then, the switch $S_{2}$ is switched ON once the SOC reaches 0.1 until the end of the day. Meanwhile, the switch $S_{3}$ is switched ON when the renewable power is less than the power required by the loads, which corresponds to Mode 3. Then, when there is an excess of RE power generated, Mode 2 is activated. The same operating strategy is obtained in December when the battery bank is initially fully charged (Figure 9). For instance, at the beginning of the day, the batteries are charged, so they supply the loads with the required power. Thus, Mode 4 is activated until the renewable energy generate the power required by the load, which corresponds to Mode 6. During this day, the switch $S_{4}$ is not switch ON since the renewable sources and the batteries can generate the electrical power required by the loads.

## 4. Discussions

In Figure 3, the obtained results demonstrate that the EMS allows the control signals of the switches to be generated correctly. In fact, at the first hours of the days, during which the power generated by the RE sources is less than the power required by the loads, the battery banks are used to complete the required power by the loads. So, the switches $S_{2}$ and $S_{3}$ are switched ON, while the switch $S_{1}$ is switched OFF. Then, when the power generated by the RE sources is higher than the power required by the loads, the excess power is used to charge the battery bank. In this case, the switches $S_{1}$ and $S_{2}$ are switched ON and the switch $S_{3}$ is switched OFF. When the loads require power during the night, only the switch $S_{3}$ is switched ON. The SOC is always maintained between the limits indicated initially in the algorithm, which are SOC min = 10% and SOC max = 80%.

Figure 4 shows that the EMS also allows for the management of the operating loads. Indeed, depending on the power required, the EMS selects which load(s) should be operating. For instance, when the required power PLoads is in the interval $\left[3000\;W\;4000\;W\right]$, then Load 1 is OFF, Load 2 is OFF, Load 3 is OFF, and Load 4 is ON. Moreover, when PLoads belongs to the interval $\left[6000\;W\;7000\;W\right]$, then Load 1 is OFF, Load 2 is OFF, Load 3 is ON, and Load 4 is ON. Figure 5, Figure 6, Figure 7 and Figure 8 demonstrate the importance of the adopted constraints applied in the EMS. Indeed, it allows for the protection of the battery against deep discharge and excessive charges. Moreover, the results show the correct operation of the switches following the objectives proposed in Section 2.2.1.

## 5. Conclusions

In this paper, an energy management strategy is studied using the fuzzy logic. The EMS is tested using the measured climatic data of the Mostoles (Spain). The results show that the EMS allows one to maximize the use of the renewable power generated by the photovoltaic panels and the wind turbine. Moreover, the battery bank is used to save the RE generated in excess or not used when the RE power generated is less than the power required by the loads. Therefore, the batteries state of charge is always maintained between the SOCmin and the SOCmax. Moreover, the diesel generator is used only in the case that the renewable sources or the battery bank cannot generate the power required by the loads. In addition, the EMS also allows the load(s) operating to be deduced. For future research, the authors would like to apply the EMS designed by applying it to enhance the energetic efficiency of buildings supplied by hybrid plants and which includes thermal and electrical controllable loads.
